# Supplementary material for: Optimal tuning of weighted kNN- and diffusion-based methods for denoising single cell genomics data
Source: PLoS Comput Biol. 2021 Jan 7;17(1):e1008569. doi: 10.1371/journal.pcbi.1008569 (PMC7817019; doi:10.1371/journal.pcbi.1008569)
Supplement: S1 Section — (PDF) [file pcbi.1008569.s001.pdf]

S1 Section Implementation of Molecular cross-validation [4]

The most straightforward method of denoising data is to use a principal component analysis (PCA); a subset of principal components (PCs) which explain a desired amount of variance are identified, and the expression data is projected onto this subset. This removes the effect of low-variance, presumably noise-driven components. The choice of how many PCs to retain greatly influences the structure of the data and the amount of remaining biological variation. DEWÄKSS utilizes PCA only to constructing the kNN-G; after the graph has been constructed denoising proceeds using the full expression matrix.

We explored selecting a reasonable number of PCs using the method Molecular cross-validation (MCV), presented by Batson et al. [4] (for use in projection algorithms). MCV takes in count data and partitions the individual counts randomly into two partitions, $(\boldsymbol{X}_J, \boldsymbol{X}_{J^c}) \in \mathbb{R}^{N,M}$. A normalization pipeline then normalizes each data partition individually to yield $(\bar{\boldsymbol{X}}_J, \bar{\boldsymbol{X}}_{J^c})$ and carries out PCA on $\bar{\boldsymbol{X}}_J$.

For different numbers of PCs, we project and then reconstruct $\bar{\boldsymbol{X}}_J$ and then calculate the Mean Squared Error (MSE) between the reconstruction and $\bar{\boldsymbol{X}}_{J^c}$. This is done as follows: let $\bar{\boldsymbol{X}}_J$ be the normalized expression matrix and $\boldsymbol{V}$ be the orthogonal matrix with columns as principal vectors. Then the mapping $\boldsymbol{Z} = \bar{\boldsymbol{X}}_{J^c}\boldsymbol{V}$ gives the projected features $\boldsymbol{Z}$ that we can map back to $\bar{\boldsymbol{X}}_J = \boldsymbol{Z}\boldsymbol{V}^T$. Using all columns of $\boldsymbol{V}$ will map $\bar{\boldsymbol{X}}_J \rightarrow \bar{\boldsymbol{X}}_J$; however, using a subset $\boldsymbol{V}_K$ of the PCs we get

$$\check{\boldsymbol{X}} = \bar{\boldsymbol{X}}_J \boldsymbol{V}_{1:k} \boldsymbol{V}_{1:k}^T \tag{S19}$$

which we can use to calculate the MSE:

$$\text{MSE} = \left\| \check{\boldsymbol{X}} - \bar{\boldsymbol{X}}_{J^c} \right\|. \tag{S20}$$

We can calculate the MSE for each number of PCs $k \in [1, K]$ and find k that corresponds to the minimum MSE:

$$k^* = \arg\min_k \left\| \boldsymbol{X}_J \boldsymbol{V}_{1:k} \boldsymbol{V}_{1:k}^T - \boldsymbol{X}_{J^c} \right\| \tag{S21}$$

We applied this algorithm using *TruncatedSVD* [32] to perform PCA and reconstruct the input matrix using the operation in equation 21.
