# Supplementary material for: Optimal tuning of weighted kNN- and diffusion-based methods for denoising single cell genomics data
Source: PLoS Comput Biol. 2021 Jan 7;17(1):e1008569. doi: 10.1371/journal.pcbi.1008569 (PMC7817019; doi:10.1371/journal.pcbi.1008569)
Supplement: S1 Table — (PDF) [file pcbi.1008569.s002.pdf]

Method	Dataset	n cells	n genes	time (min)	time (s)
DeepImpute	Hrvatin et al. [19]	64883	18169	17.5888	1055.33
DEWÄKSS	Hrvatin et al. [19]	64883	18169	107.164	6429.85
MAGIC	Hrvatin et al. [19]	64883	18169	9.37999	562.799
DrImpute	EMT_MAGIC[10]	7523	18259	50.6583	3039.5
DeepImpute	EMT_MAGIC[10]	7523	18259	5.39482	323.689
DEWÄKSS	EMT_MAGIC[10]	7523	18259	12.3992	743.953
MAGIC	EMT_MAGIC[10]	7523	18259	0.696018	41.7611
SAVER	EMT_MAGIC[10]	7523	18259	65.3612	3921.68
DrImpute	Jackson et al. [21]	38225	5863	182.982	10978.9
DeepImpute	Jackson et al. [21]	38225	5863	8.65502	519.301
DEWÄKSS	Jackson et al. [21]	38225	5863	43.7966	2627.8
MAGIC	Jackson et al. [21]	38225	5863	3.29837	197.902
DrImpute	Paul et al. [31]	2730	9504	5.1723	310.338
DeepImpute	Paul et al. [31]	2730	9504	0.96728	58.0368
DEWÄKSS	Paul et al. [31]	2730	9504	3.49939	209.963
MAGIC	Paul et al. [31]	2730	9504	0.0931717	5.5903
SAVER	Paul et al. [31]	2730	9504	27.2776	1636.66
DrImpute	Zeisel et al. [44]	3005	16450	2.9967	179.802
DeepImpute	Zeisel et al. [44]	3005	16450	4.55035	273.021
DEWÄKSS	Zeisel et al. [44]	3005	16450	6.3853	383.118
MAGIC	Zeisel et al. [44]	3005	16450	0.16204	9.72242
SAVER	Zeisel et al. [44]	3005	16450	98.2859	5897.16

S1 Table: Computational performance results for the tested methods.
