# Supplementary material for: Optimal tuning of weighted kNN- and diffusion-based methods for denoising single cell genomics data
Source: PLoS Comput Biol. 2021 Jan 7;17(1):e1008569. doi: 10.1371/journal.pcbi.1008569 (PMC7817019; doi:10.1371/journal.pcbi.1008569)
Supplement: S2 Table — The Gold Standard (GS) is knockout strains collected from the bulk deleteome data [39]. (PDF) [file pcbi.1008569.s003.pdf]

metric	gene<br>method	dal80	dal81	dal82	gat1	gcn4	gln3	gzf3	rtg1	rtg3	stp1	stp2
aupr	DEWAKSS	0.000	0.262	0.000	0.000	2.913e-02	0.408	0.000	0.162	0.163	0.525	0.000
	DeepImpute	0.000	0.231	0.326	1.706e-04	1.192e-02	0.325	0.077	0.116	0.181	0.501	0.050
	DrImpute	0.000	0.217	0.135	5.482e-04	1.243e-02	0.289	0.039	0.161	0.136	0.500	0.040
	MAGIC	0.000	0.000	0.000	0.000	1.965e-02	0.325	0.000	0.046	0.084	0.504	0.000
	SAVER	1.706e-04	0.270	0.251	0.000	3.342e-02	0.374	0.056	0.212	0.213	0.544	0.152
	preprocessed	0.000	0.264	0.326	1.706e-04	2.291e-02	0.352	0.144	0.190	0.197	0.528	0.349
auroc	DEWAKSS	5.000e-01	0.579	0.500	5.000e-01	5.233e-01	0.614	0.500	0.702	0.618	0.533	0.500
	DeepImpute	5.000e-01	0.525	0.550	4.999e-01	5.110e-01	0.538	0.510	0.646	0.574	0.506	0.543
	DrImpute	5.000e-01	0.526	0.606	8.444e-01	4.979e-01	0.525	0.552	0.770	0.618	0.499	0.522
	MAGIC	5.000e-01	0.500	0.500	5.000e-01	5.432e-01	0.534	0.500	0.525	0.561	0.511	0.500
	SAVER	4.597e-01	0.585	0.550	5.000e-01	5.810e-01	0.588	0.531	0.691	0.646	0.560	0.653
	preprocessed	5.000e-01	0.519	0.550	5.001e-01	5.070e-01	0.528	0.521	0.650	0.571	0.508	0.506
avg_pr	DEWAKSS	0.000	0.262	0.000	0.000	2.969e-02	0.409	0.000	0.166	0.164	0.525	0.000
	DeepImpute	0.000	0.198	0.102	3.411e-04	9.468e-03	0.297	0.015	0.105	0.118	0.493	0.041
	DrImpute	0.000	0.218	0.102	1.096e-03	1.593e-02	0.290	0.041	0.163	0.137	0.501	0.041
	MAGIC	0.000	0.000	0.000	0.000	1.069e-02	0.326	0.000	0.046	0.083	0.504	0.000
	SAVER	3.411e-04	0.267	0.102	0.000	3.306e-02	0.372	0.056	0.216	0.204	0.540	0.153
	preprocessed	0.000	0.195	0.102	3.412e-04	1.516e-02	0.296	0.044	0.150	0.123	0.501	0.036
jaccard	DEWAKSS	0.000	0.135	0.000	0.000	8.091e-03	0.282	0.000	0.032	0.079	0.350	0.000
	DeepImpute	0.000	0.017	0.100	0.000	0.000	0.108	0.000	0.093	0.062	0.111	0.006
	DrImpute	0.000	0.113	0.100	0.000	7.759e-03	0.281	0.021	0.023	0.065	0.428	0.012
	MAGIC	0.000	0.000	0.000	0.000	9.773e-03	0.194	0.000	0.017	0.061	0.337	0.000
	SAVER	0.000	0.114	0.100	0.000	2.066e-02	0.238	0.036	0.056	0.125	0.324	0.117
	preprocessed	0.000	0.016	0.100	0.000	1.667e-02	0.036	0.043	0.139	0.064	0.025	0.006
mcc	DEWAKSS	0.000	0.113	0.000	0.000	-9.286e-04	0.111	0.000	0.085	0.088	0.052	0.000
	DeepImpute	0.000	0.065	0.316	-2.413e-04	-5.300e-03	0.034	-0.001	0.157	0.137	-0.003	0.052
	DrImpute	0.000	0.034	0.316	-2.413e-04	-4.996e-03	0.043	0.103	0.074	0.061	-0.012	0.073
	MAGIC	0.000	0.000	0.000	0.000	9.125e-03	0.052	0.000	0.009	0.035	0.012	0.000
	SAVER	-2.413e-04	0.130	0.316	0.000	3.271e-02	0.108	0.085	0.136	0.170	0.067	0.241
	preprocessed	0.000	0.058	0.316	-2.413e-04	4.529e-02	0.033	0.169	0.237	0.141	0.022	0.052
predicted DEGs	DEWAKSS	0	703	0	0	570	3605	0	1749	1847	2870	0
	DeepImpute	0	37	1	1	18	715	1	104	65	738	2
	DrImpute	0	1024	1	1	1116	5322	2	3755	3831	4525	4
	MAGIC	0	0	0	0	3150	1738	0	3321	3719	2933	0
	SAVER	1	417	1	0	194	2040	11	787	654	2453	49
	preprocessed	0	38	1	1	8	175	3	69	67	132	2
GS DEGs	bulk data	2	1032	10	2	53	1647	46	95	330	2908	170

S2 Table: All metrics computed for benchmarking deferentially expressed genes (DEGs), Section 2.6. The Gold Standard (GS) is knockout strains collected from the bulk deleteome data[22].
