# Supplementary material for: Optimal tuning of weighted kNN- and diffusion-based methods for denoising single cell genomics data
Source: PLoS Comput Biol. 2021 Jan 7;17(1):e1008569. doi: 10.1371/journal.pcbi.1008569 (PMC7817019; doi:10.1371/journal.pcbi.1008569)
Supplement: S1 Fig — We use the R code provided in Tian et al. [43] to apply all normalization methods that we can successfully run on the RNAmix_CEL-seq2 dataset. We also apply our FTT-based preprocessing method on the data. We use the postprocessing and plotting scripts in [43] to generate plots for our analysis. (PDF) [file pcbi.1008569.s004.pdf]

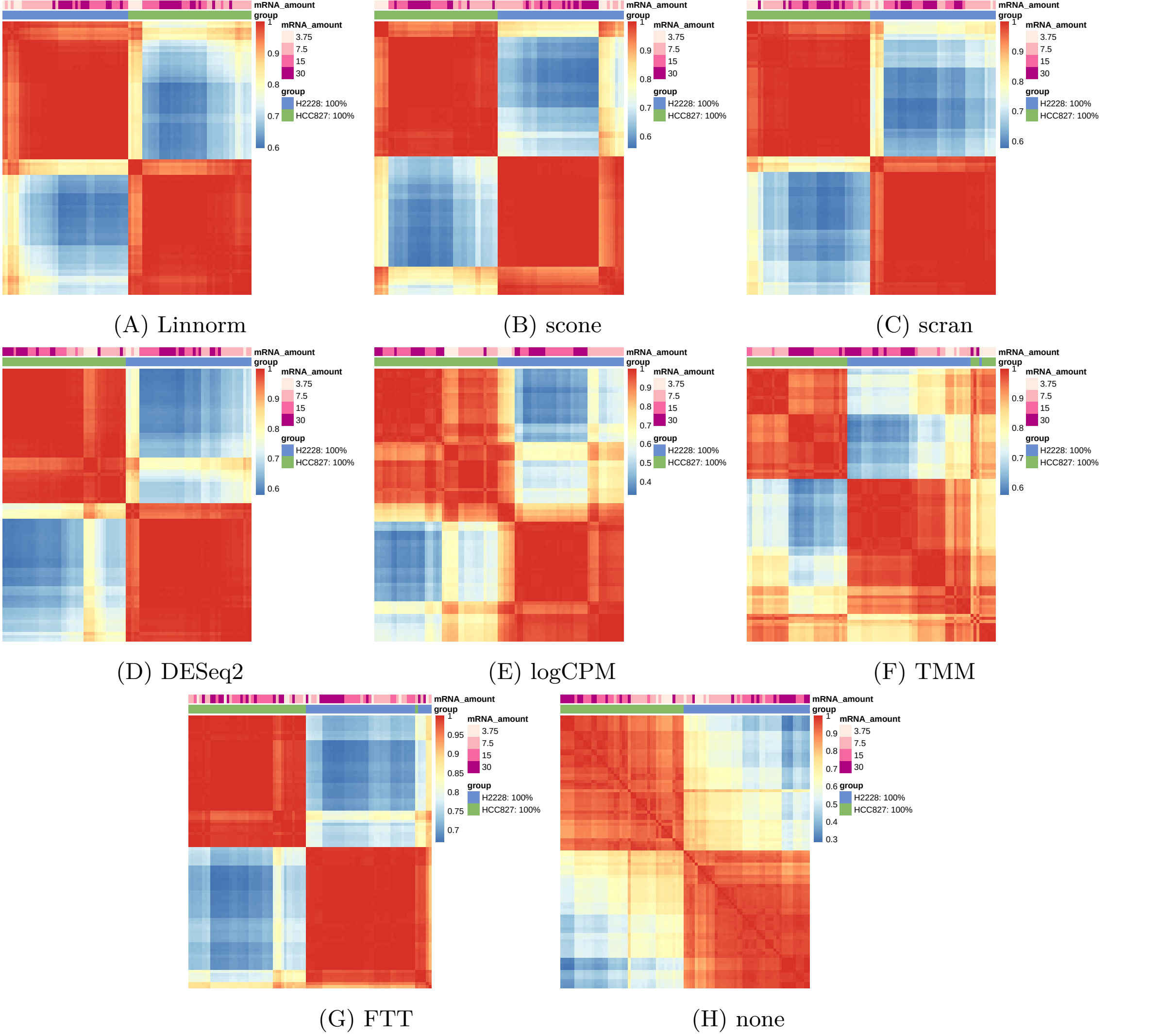

S1 Fig: Heatmaps of normalized and denoised data on the RNAmix_CEL-seq2 dataset [36]. We use the R code provided in Tian et al. [37] to apply all normalization methods that we can successfully run on the RNAmix_CEL-seq2 dataset. We also apply our FTT-based preprocessing method on the data. We use the postprocessing and plotting scripts in [37] to generate plots for our analysis.
