# Supplementary material for: Optimal tuning of weighted kNN- and diffusion-based methods for denoising single cell genomics data
Source: PLoS Comput Biol. 2021 Jan 7;17(1):e1008569. doi: 10.1371/journal.pcbi.1008569 (PMC7817019; doi:10.1371/journal.pcbi.1008569)
Supplement: S2 Fig — The dataset contains 33 annotated celltypes in 48267 cells. a) Clustering performance metric computed against cell type clusters. Clusters were inferred with the Leiden algorithm [19]. The number at the bottom of each bar represents the inferred number of clusters. The Leiden algorithm’s resolution parameter r was set to 0.5 for MAGIC, 2 for pp and DeepImpute and 4 for DrImpute and SAVER to reduce or increase number of clusters, respectively, to comparative numbers given the previously annotated clusters. b) Silhouette score computed on 2 UMAP components using the 33 predefined cell type clusters. The input parameters to the umap algorithm is annotated with x and y labels of each panels row and column. c) Silhouette score computed on a varying numbers of PC components using the 33 predefined cell clusters. The number of PCs used corresponds to the number on the upper edge of the graph. (PDF) [file pcbi.1008569.s005.pdf]

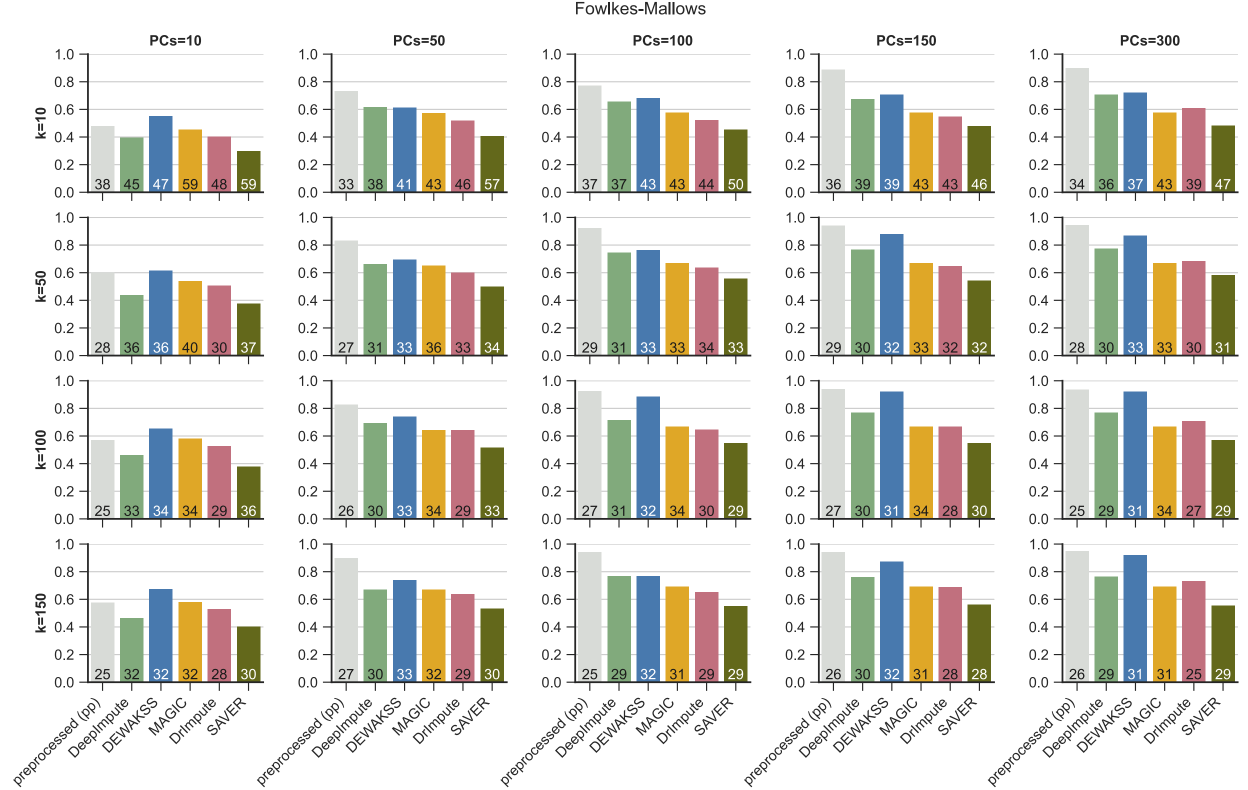

(A) Clustering performance metric computed against cell type clusters. Clusters were inferred with the Leiden algorithm[38]. The number at the bottom of each bar represents the inferred number of clusters. The Leiden algorithm's resolution parameter r was set to 0.5 for MAGIC, 2 for pp and DeepImpute and 4 for DrImpute and SAVER to reduce or increase number of clusters, respectively, to comparative numbers given the previously annotated clusters.

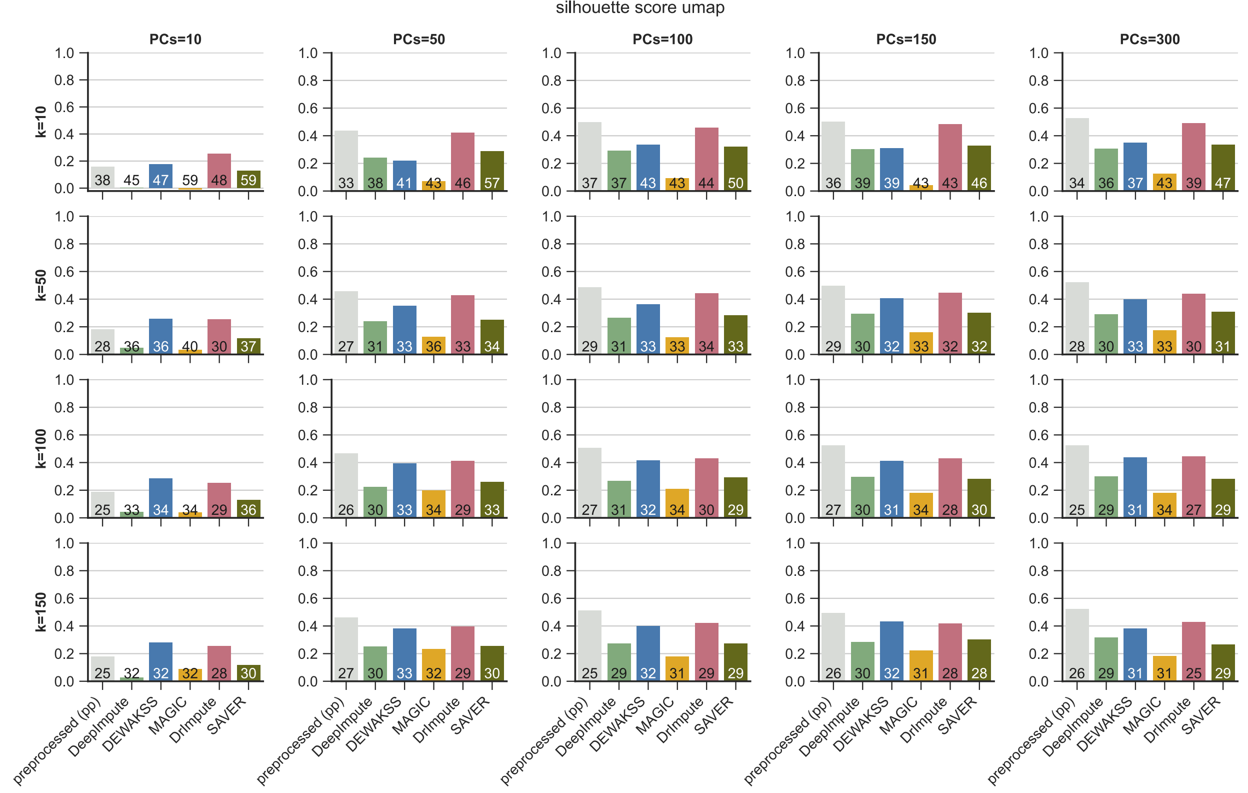

(B) Silhouette score computed on 2 UMAP components using the 33 predefined cell type clusters. The input parameters to the umap algorithm is annotated with x and y labels of each panels row and column.

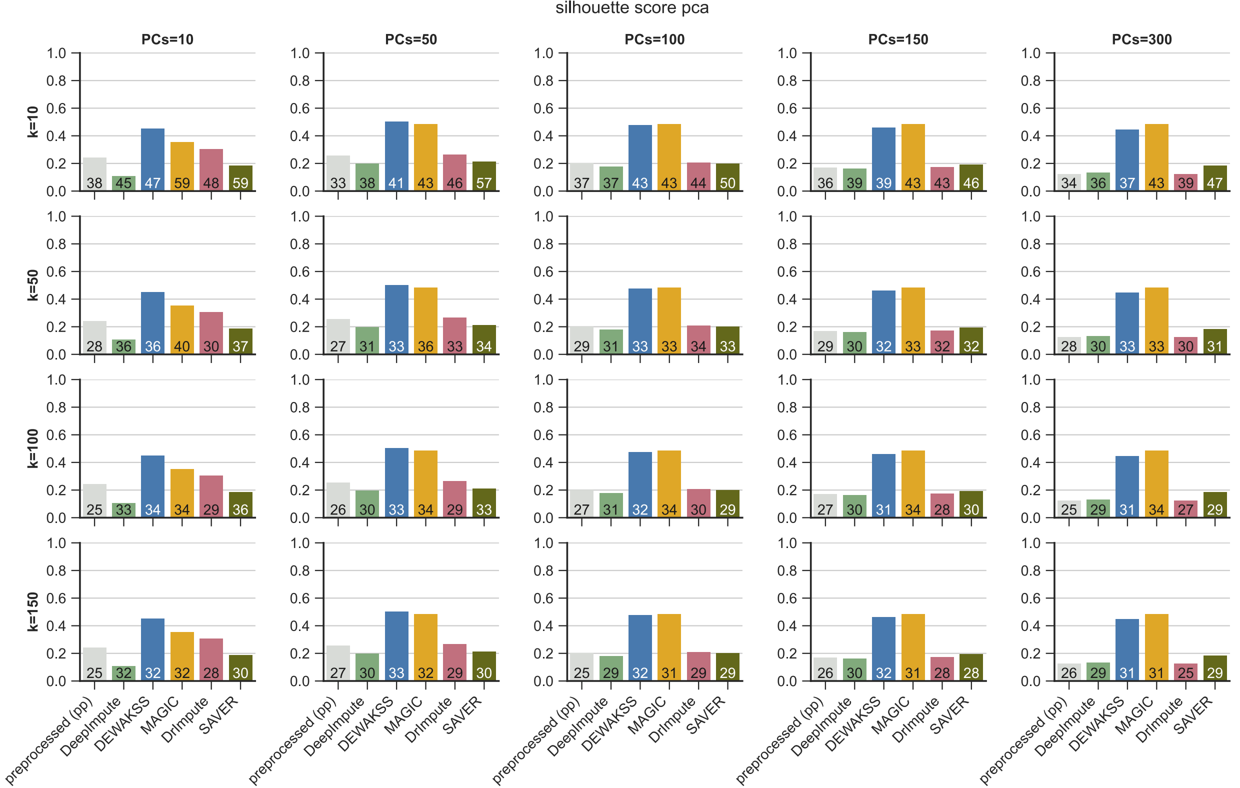

(C) Silhouette score computed on a varying numbers of PC components using the 33 predefined cell clusters. The number of PCs used corresponds to the number on the upper edge of the graph.

S2 Fig: Denoising celltype annotated data from Hrvatin et al. [19]. The dataset contains 33 annotated celltypes in 48267 cells.
