# Supplementary material for: Optimal tuning of weighted kNN- and diffusion-based methods for denoising single cell genomics data
Source: PLoS Comput Biol. 2021 Jan 7;17(1):e1008569. doi: 10.1371/journal.pcbi.1008569 (PMC7817019; doi:10.1371/journal.pcbi.1008569)
Supplement: S3 Fig — Explained variance η2 for each component Σ (top row) and cumulative sum of η2 for each component Σ (bottom row). Each colored line indicates the data denoised with a specific set of parameters for the algorithms DEWÄKSS and MAGIC. X indicates the data without denoising. MAGIC truncates the number of possible components to the number of PCs used in the algorithm, which here equals 100. The right column only shows the first 100 components for the respective η2 and cumsum(η2). MAGICd1 is removed from the top right-hand figure because it compresses the other lines. The explained variance is computed through the singular value decomposition and singular values lower than the numerical precision threshold are considered equal to 0 and removed. This threshold is determined by the criterion σi ≤ σ1 × max(i, j) × ϵ, where i, j are the data dimensions and ϵ is the machine precision (numpy matrix_rank). (PDF) [file pcbi.1008569.s006.pdf]

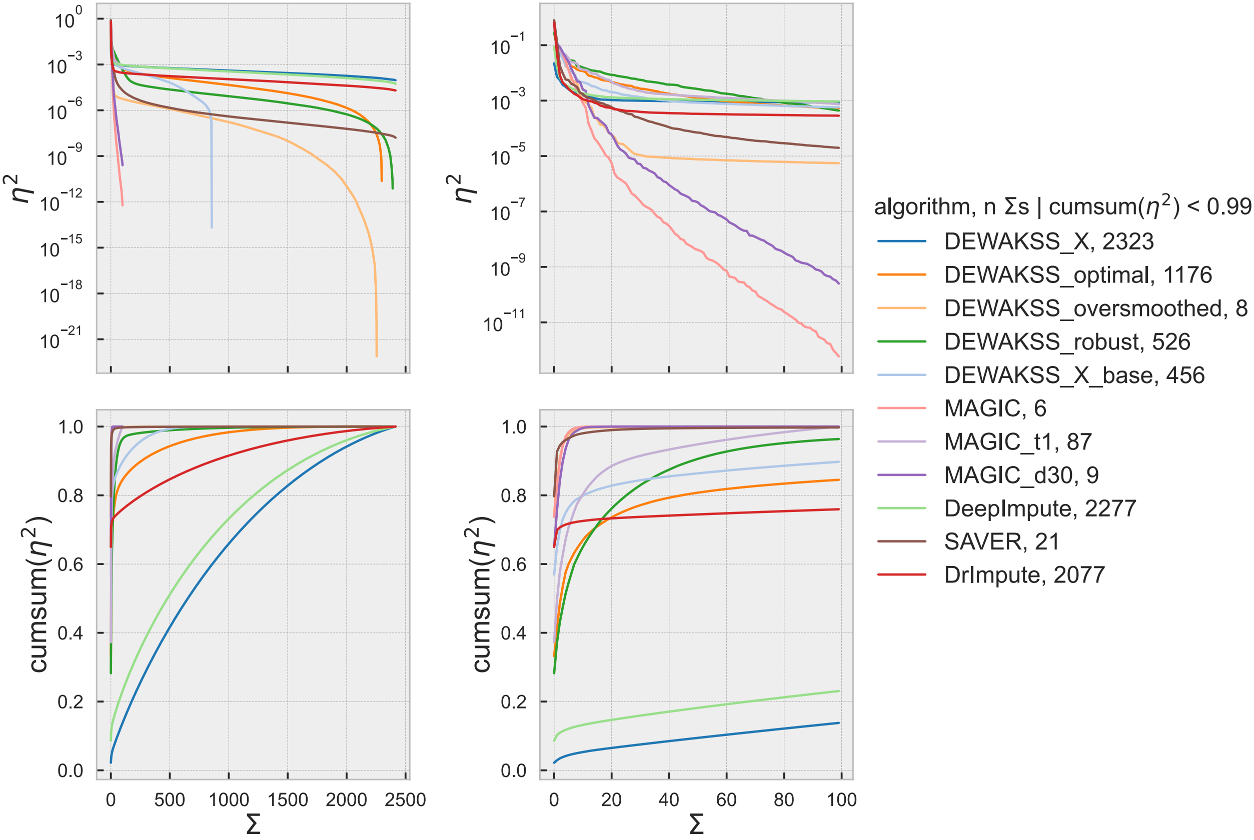

S3 Fig: BM data [31]. Explained variance η^2 for each component Σ (top row) and cumulative sum of η^2 for each component Σ (bottom row). Each colored line indicates the data denoised with a specific set of parameters for the algorithms DEWÄKSS and MAGIC. X indicates the data without denoising. MAGIC truncates the number of possible components to the number of PCs used in the algorithm, which here equals 100. The right column only shows the first 100 components for the respective η^2 and $cumsum(\eta^2)$. MAGICd1 is removed from the top right-hand figure because it compresses the other lines. The explained variance is computed through the singular value decomposition and singular values lower than the numerical precision threshold are considered equal to 0 and removed. This threshold is determined by the criterion $\sigma_i \leq \sigma_1 \times \max(i, j) \times \epsilon$, where i, j are the data dimensions and ϵ is the machine precision (numpy matrix_rank).
