# Supplementary material for: Optimal tuning of weighted kNN- and diffusion-based methods for denoising single cell genomics data
Source: PLoS Comput Biol. 2021 Jan 7;17(1):e1008569. doi: 10.1371/journal.pcbi.1008569 (PMC7817019; doi:10.1371/journal.pcbi.1008569)
Supplement: S4 Fig — A) The numbers of components needed to explain 99% and 90% of the variance in the data for different methods and hyperparameter values. MAGIC is run with 3 settings: default, t1 = one iteration, and “dewakss”, using the optimal configuration found by DEWÄKSS. DEWÄKSS is run with 4 different settings: (i) optimal, as found by iterating over a range of hyperparameters (panel B and S6(B) Fig), (ii) oversmoothed, by running to i = 4 iterations, (iii) robust, i.e., using a different set of hyperparameters (k = 100, PCs = 23 selected as in S1 Section, i = iminMSE) and (iv) X base (k = 100, i = iminMSE), using normalized expression values instead of principal components as input to the kNN-G algorithm. B) The lowest MSE over all iteration values as a function of each DEWÄKSS parameter configuration, using connectivity graphs in the left plot and distances in the right plot. The lowest MSE configuration is found using distances with 100 PCs and k = 100 neighbors. (PDF) [file pcbi.1008569.s007.pdf]

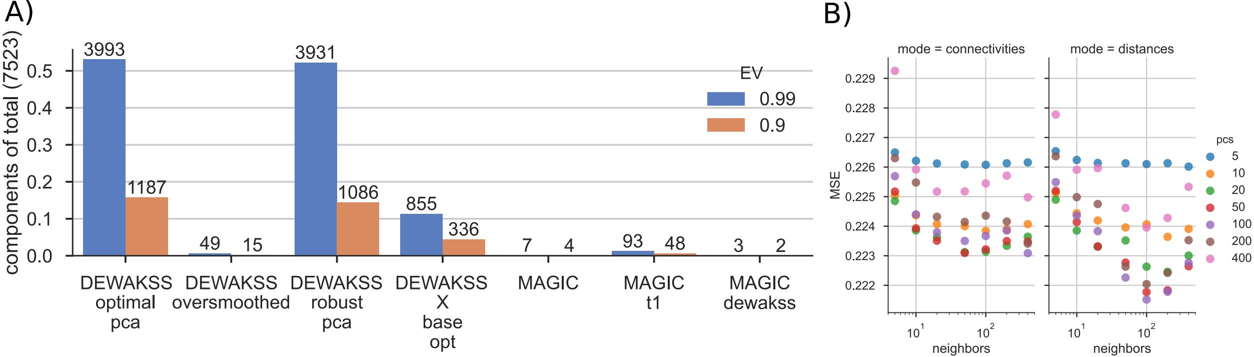

S4 Fig: Epithelial-to-mesenchymal transition (EMT) data denoising. A) The numbers of components needed to explain 99% and 90% of the variance in the data for different methods and hyperparameter values. MAGIC is run with 3 settings: default, t1 = one iteration, and "dewakss", using the optimal configuration found by DEWÄKSS. DEWÄKSS is run with 4 different settings: (i) optimal, as found by iterating over a range of hyperparameters (panel B and S6 FigB), (ii) oversmoothed, by running to $i = 4$ iterations, (iii) robust, i.e., using a different set of hyperparameters ($k = 100$, $PCs = 23$ selected as in S1 Section, $i = i_{\min MSE}$) and (iv) X base ($k = 100$, $i = i_{\min MSE}$), using normalized expression values instead of principal components as input to the kNN-G algorithm. B) The lowest MSE over all iteration values as a function of each DEWÄKSS parameter configuration, using connectivity graphs in the left plot and distances in the right plot. The lowest MSE configuration is found using distances with 100 PCs and $k = 100$ neighbors.
