# Supplementary material for: Optimal tuning of weighted kNN- and diffusion-based methods for denoising single cell genomics data
Source: PLoS Comput Biol. 2021 Jan 7;17(1):e1008569. doi: 10.1371/journal.pcbi.1008569 (PMC7817019; doi:10.1371/journal.pcbi.1008569)
Supplement: S5 Fig — The optimal number of PCs and neighbors is independent of the number of diffusion steps. (PDF) [file pcbi.1008569.s008.pdf]

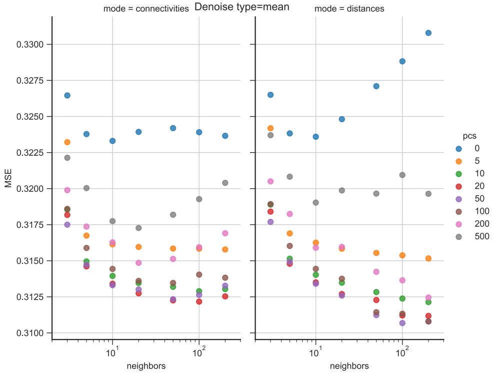

(A) Paul et al. [31] BM dataset.

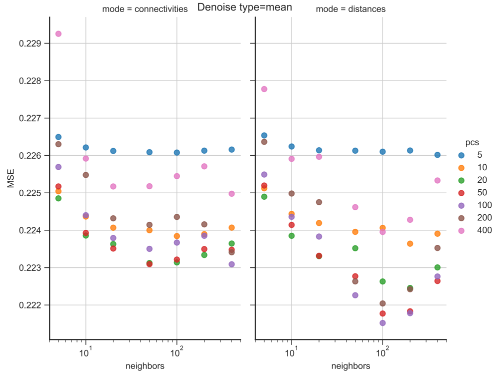

(B) van Dijk et al. [10] EMT dataset.

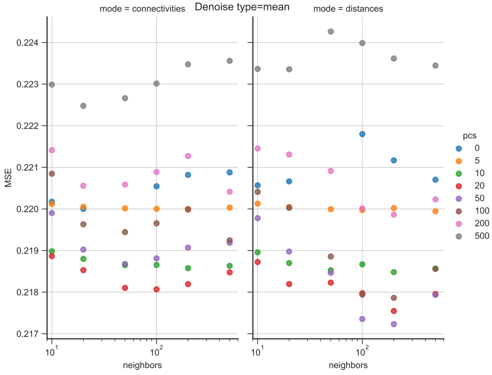

(C) Jackson et al. [21] YPD growth condition

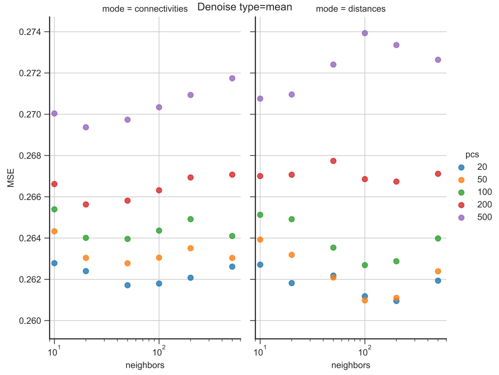

(D) Jackson et al. [21] YPDRapa growth condition

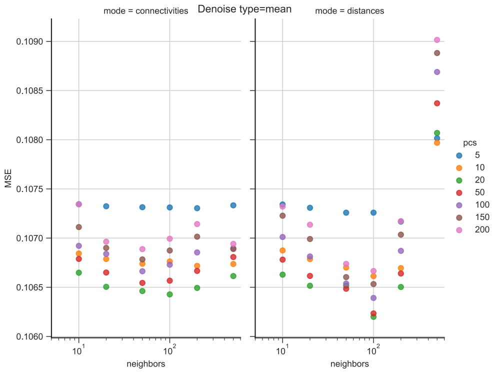

(E) La Manno et al. [25] hgForebrainGlut

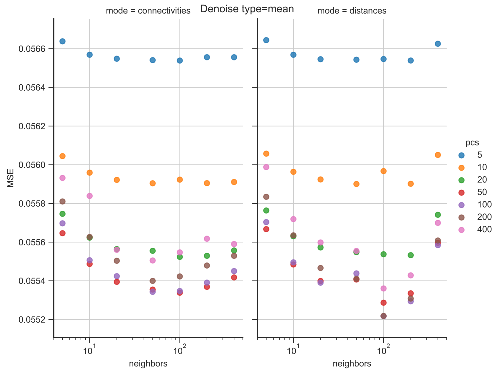

(F) La Manno et al. [25] DentateGyrus

S5 Fig: Optimal hyperparameter search for single-cell datasets. The optimal number of PCs and neighbors is independent of the number of diffusion steps.
