# Supplementary material for: Optimal tuning of weighted kNN- and diffusion-based methods for denoising single cell genomics data
Source: PLoS Comput Biol. 2021 Jan 7;17(1):e1008569. doi: 10.1371/journal.pcbi.1008569 (PMC7817019; doi:10.1371/journal.pcbi.1008569)
Supplement: S6 Fig — Each figure shows the algorithm, using distances (top row) and connectivities (bottom row). Each panel is scaled to the maximum number of observed iterations (x-axis) for any configuration run, with the objective function value MSE on the y-axis. The colored lines indicate the number of PCs used as input in the kNN-G distance computation. Each column corresponds to the initial number of neighbors k used for constructing the kNN-G. (PDF) [file pcbi.1008569.s009.pdf]

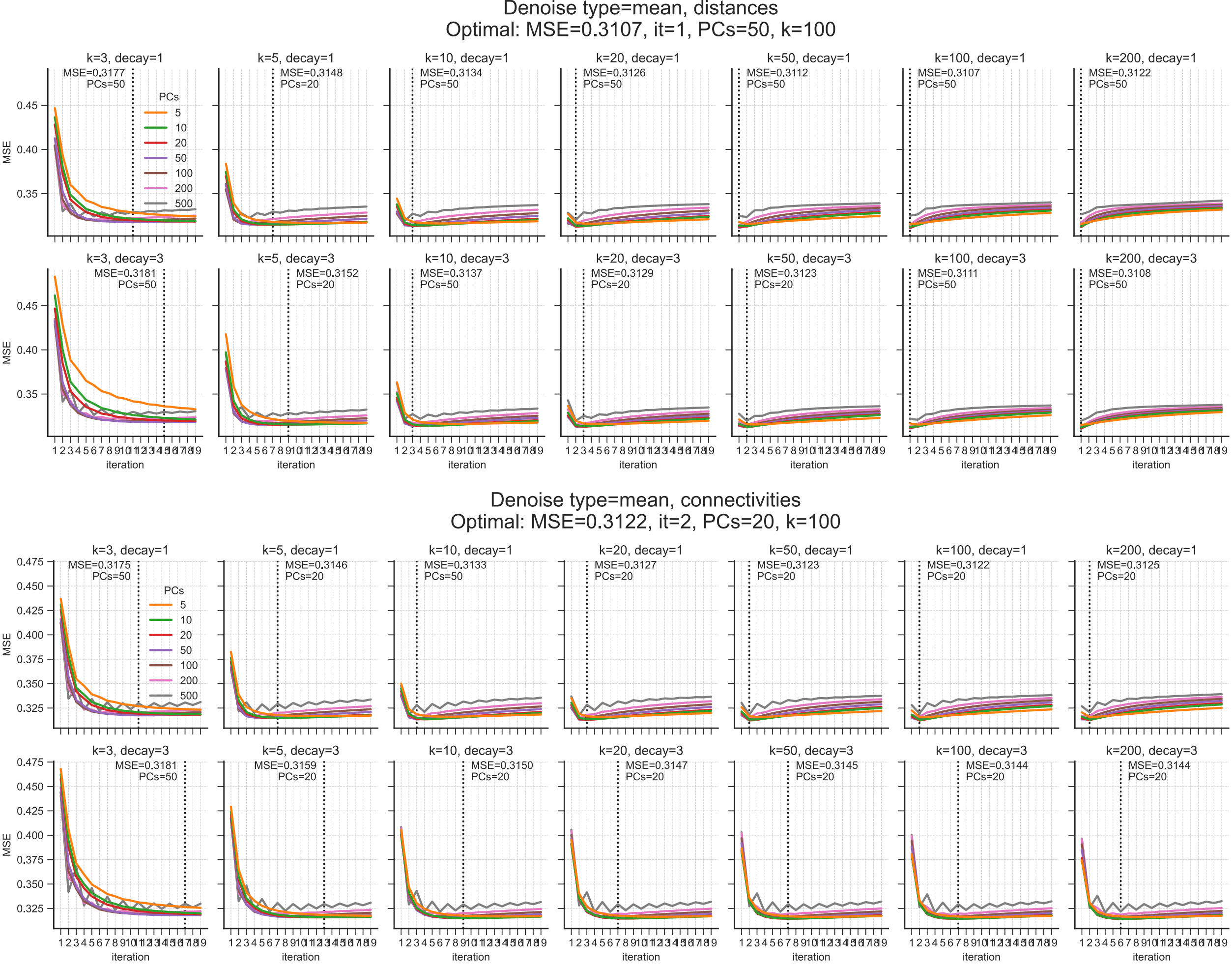

(A) BM data. DEWÄKSS objective function values, mean square error, MSE during optimization of a range of hyperparameters, number of principal components (PCs) $= \{5, 10, 20, 50, 100, 200, 500\}$, number of initial neighbors (k) $= \{3, 5, 10, 20, 50, 100, 200\}$ and decay $= \{1, 3\}$. Optimal settings are found to be 50 PCs, decay $= 1$, and $k = 100$ with MSE $= 0.3107$

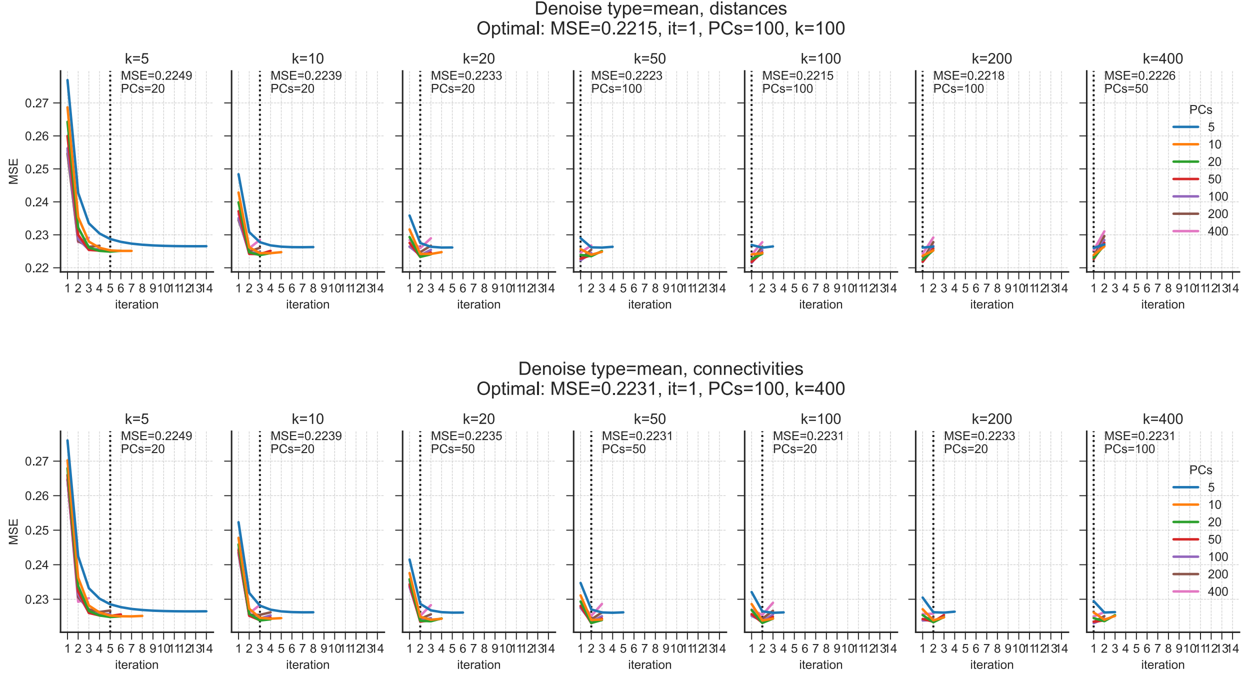

(B) EMT data. DEWÄKSS objective function values, mean square error, MSE during optimization of a range of hyperparameters, number of principal components (PCs) = 5, 10, 20, 50, 100, 200, 400}, number of initial neighbors (k) = $\{5, 10, 20, 50, 100, 200, 400\}$. The top row uses distances on the kNN-G and the bottom row uses connectivites of the kNN-G. Optimal settings are found to be 100PCs, $k = 100$ using distances with MSE = 0.2215. The algorithm's option to stop after the minimum is found is used here to stop the algorithm once the optimum (min MSE) is found.

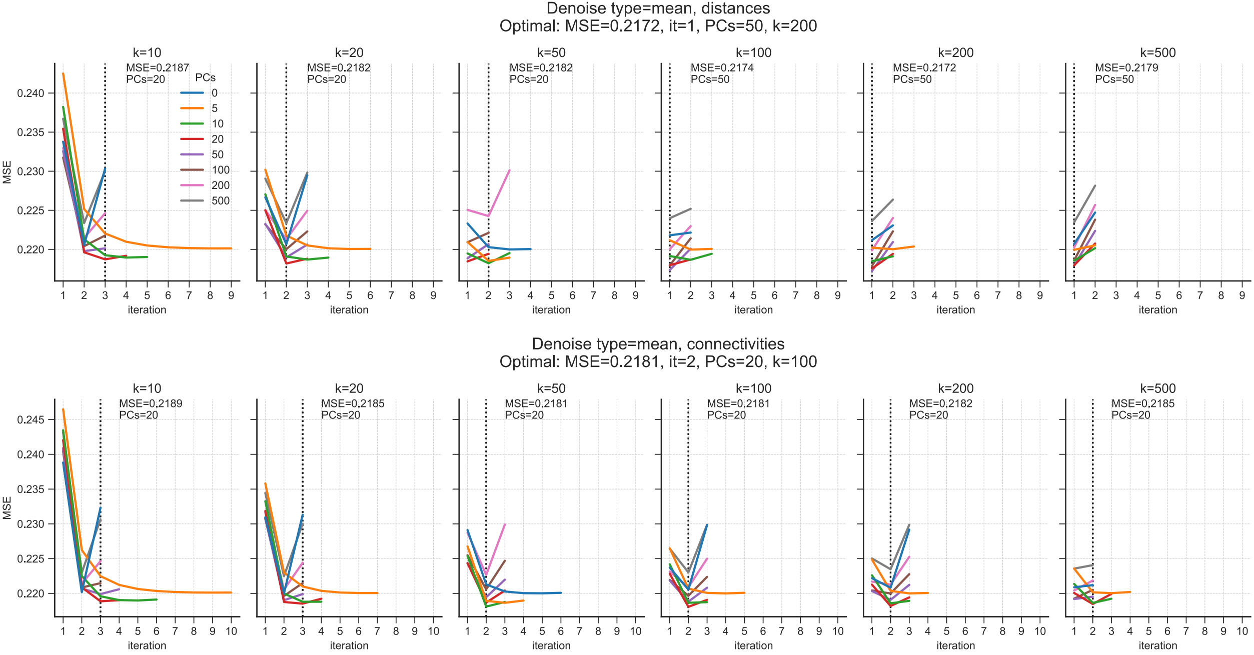

(C) Jackson et al. [21] YPD growth condition

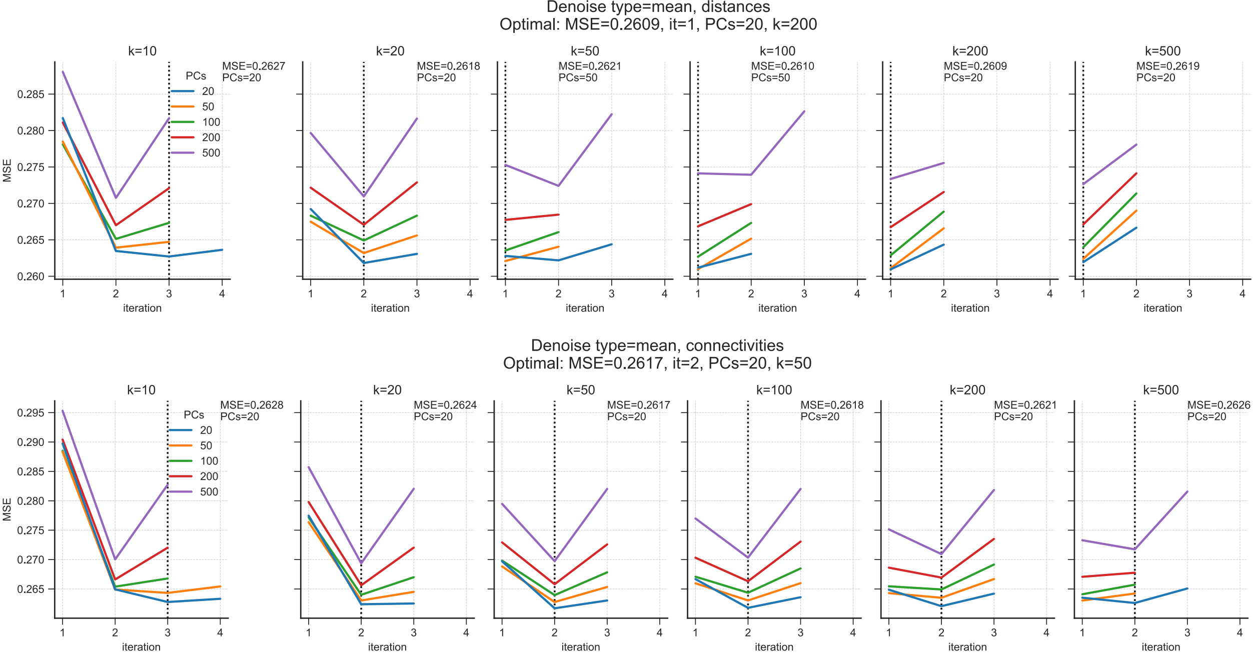

(D) Jackson et al. [21] YPDRapa growth condition

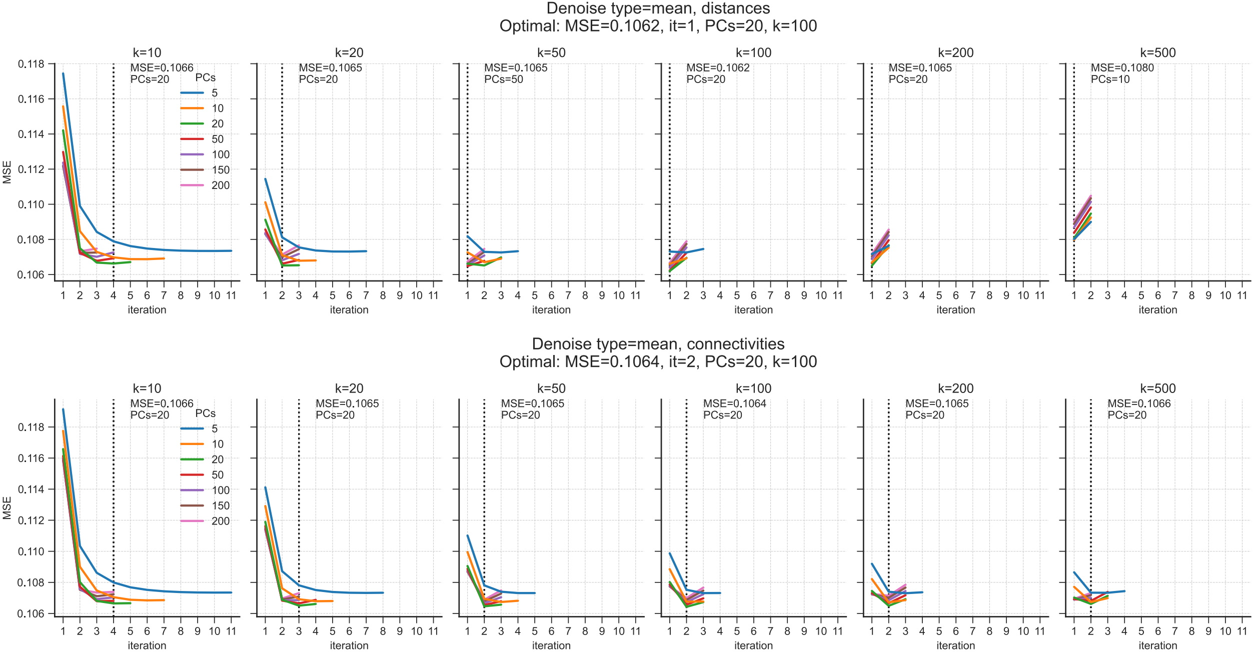

(E) La Manno et al. [25] hgForebrainGlut

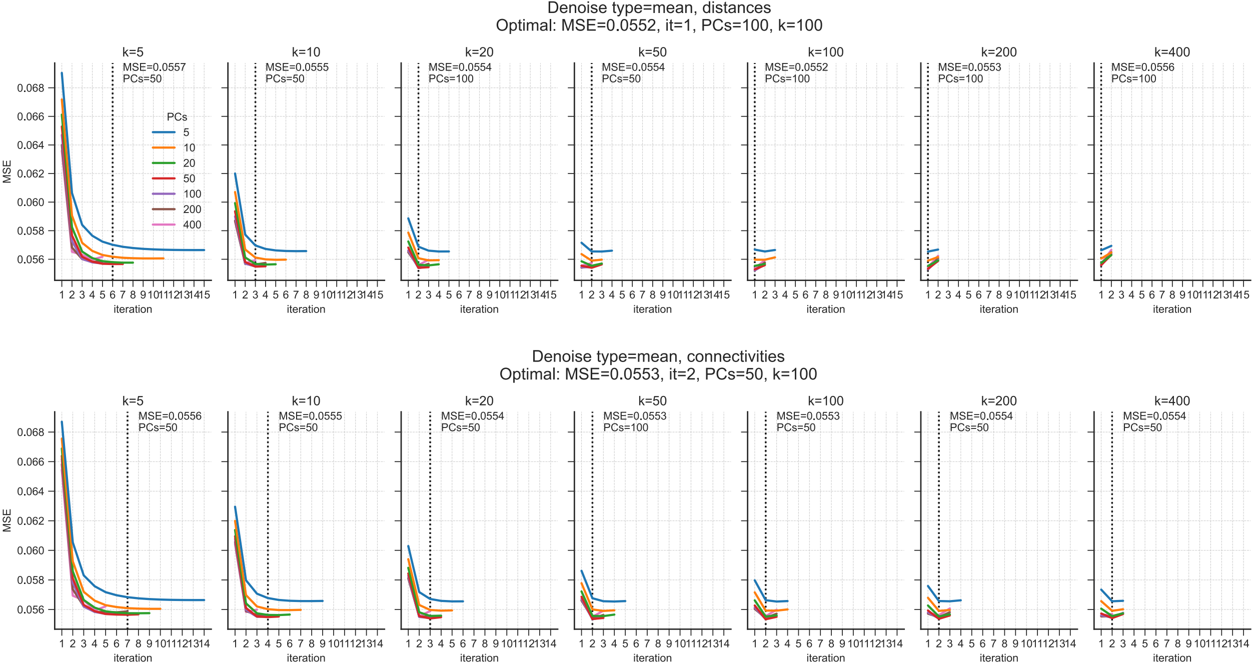

(F) La Manno et al. [25] DentateGyrus

S6 Fig: Optimal hyperparameter search for single-cell datasets. Each figure shows the algorithm, using distances (top row) and connectivities (bottom row). Each panel is scaled to the maximum number of observed iterations (x-axis) for any configuration run, with the objective function value MSE on the y-axis. The colored lines indicate the number of PCs used as input in the kNN-G distance computation. Each column corresponds to the initial number of neighbors k used for constructing the kNN-G.
