# Supplementary material for: Optimal tuning of weighted kNN- and diffusion-based methods for denoising single cell genomics data
Source: PLoS Comput Biol. 2021 Jan 7;17(1):e1008569. doi: 10.1371/journal.pcbi.1008569 (PMC7817019; doi:10.1371/journal.pcbi.1008569)
Supplement: S7 Fig — This makes the objective function self referential and breaks the requirement setup by the noise2self principle. At the second diffusion step it becomes possible for cells to start influencing their own objective and therefore minimizes the objective function. This is akin to overfitting where each datapoint is best predicted by itself. This would encourage fewer neighbours as it will mirror the datapoint itself better. Note that diffusion turns out to be not needed and the methods converge for large enough k of initial neighbours. (PDF) [file pcbi.1008569.s010.pdf]

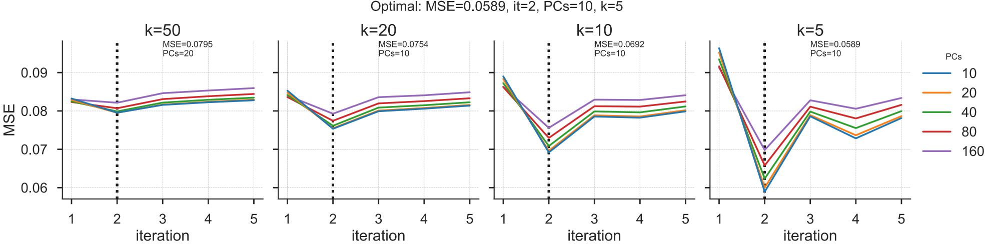

S7 Fig: Modified DEWÄKSS algorithm to not reset diagonal elements to 0 after each diffusion run. This makes the objective function self referential and breaks the requirement setup by the noise2self principle. At the second diffusion step it becomes possible for cells to start influencing their own objective and therefore minimizes the objective function. This is akin to overfitting where each datapoint is best predicted by itself. This would encourage fewer neighbours as it will mirror the datapoint itself better. Note that diffusion turns out to be not needed and the methods converge for large enough k of initial neighbours.
