# Supplementary material for: Optimal tuning of weighted kNN- and diffusion-based methods for denoising single cell genomics data
Source: PLoS Comput Biol. 2021 Jan 7;17(1):e1008569. doi: 10.1371/journal.pcbi.1008569 (PMC7817019; doi:10.1371/journal.pcbi.1008569)
Supplement: S8 Fig — MCV is here used to find optimal number of PCs to linearly project the data into. MCV with PCA shows that a linear projection on the data is best described by 13 components. (PDF) [file pcbi.1008569.s011.pdf]

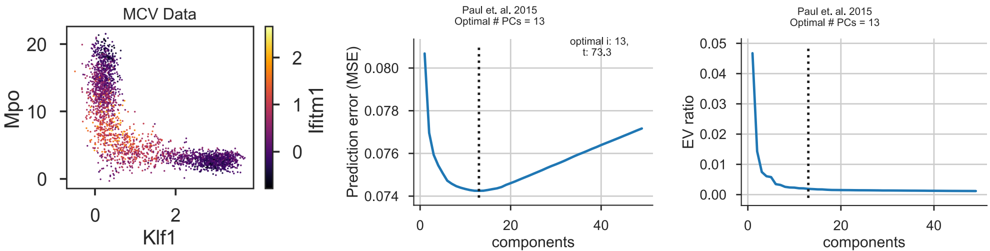

S8 Fig: Molecular cross validation (MCV) on Paul et al. [31]. MCV is here used to find optimal number of PCs to linearly project the data into. MCV with PCA shows that a linear projection on the data is best described by 13 components.
