# Supplementary material for: Optimal tuning of weighted kNN- and diffusion-based methods for denoising single cell genomics data
Source: PLoS Comput Biol. 2021 Jan 7;17(1):e1008569. doi: 10.1371/journal.pcbi.1008569 (PMC7817019; doi:10.1371/journal.pcbi.1008569)
Supplement: S9 Fig — Top panel is delta AUROC between bulk DEGs and single cell DEGs ordered by adjusted p-value. Bottom panel is delta Jaccard index for single-cell DEGs at FDR = 0.01 for each separate deletion strain. preprocessed is count normalized and log-transformed with no denoising method. Delta is taken between denoised and preprocessed. (PDF) [file pcbi.1008569.s012.pdf]

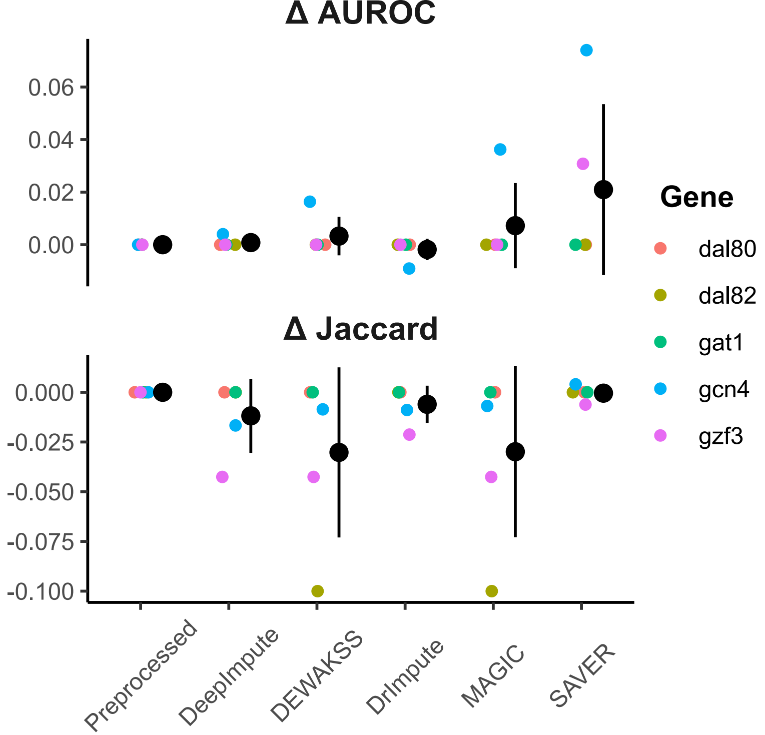

S9 Fig: Differentially expressed genes (DEGs) between bulk and single cell data for deletion strains with few DEGs (< 63, 1% of yeast genome). Top panel is delta AUROC between bulk DEGs and single cell DEGs ordered by adjusted p-value. Bottom panel is delta Jaccard index for single-cell DEGs at FDR $= 0.01$ for each separate deletion strain. *preprocessed* is count normalized and log-transformed with no denoising method. Delta is taken between denoised and preprocessed.
