# Supplementary material for: Optimal tuning of weighted kNN- and diffusion-based methods for denoising single cell genomics data
Source: PLoS Comput Biol. 2021 Jan 7;17(1):e1008569. doi: 10.1371/journal.pcbi.1008569 (PMC7817019; doi:10.1371/journal.pcbi.1008569)
Supplement: Attachment — Submitted filename: Response to reviewer (PCOMPBIOL-D-20-00345).docx [file pcbi.1008569.s013.docx]

Dear Drs. Nie and Zhang,

Thanks very much for a very careful review of our manuscript, we have been able to address the comments raised below, and think the reviews have prompted many positive changes to the manuscript. A copy of the revised manuscript with changes highlighted as well as a clean copy has been attached to this correspondence as requested.

In particular: 1) we now compare more completely to noise2self and mathematical components of that methods and increase the clarity of discussions of this great prior work, 2) we added a much more complete set of prior methods as requested, 3) we discuss our choice of benchmarks more completely and add text to address reviewer concerns about using variance recovered as a key metric, 4) we fixed the notation and responded to comments about choice of notation in methods, 5) we added a benchmark on differential gene expression recovery, and 6) we have now added requested computational benchmarks.

We detail responses to all reviewer comments and questions below.

Thanks again for your consideration of our work !

Sincerely,

Rich and Andreas

--------------

**Reviewer #1**

**This paper presents an interesting approach to denoising single-cell RNA-seq data based on (a) construction of a kNN graph (after PCA dimensionality reduction), (b) diffusion based denoising and (c) noise2self self-supervision procedure to select hyperparameters in this procedure: (i) dimension of PCA embedding, (ii) number of neighbors in the kNN graph, and (iii, presumably) number of diffusion steps in denoising.**

**The paper is well written, with appropriate benchmarking, analysis and interpretation. The method itself provides benefits in analysis due to the ability to provide a procedure for parameter selection based on self-supervision.**

**Nonetheless it would be helpful if the manuscript addressed some issues:**

**(a) comparison with noise2self pipeline for analysis. Some of the analysis of this paper mirror analyses from the noise2self paper (eg, the comparison of marker expression in optimal denoising vs. over smoothing). With that in mind, it would be helpful to see a comparison of the proposed algorithm with noise2self.**

Although the noise2self concept is not a denoising *method* in and of itself, the molecular cross validation (MCV) framework developed by the authors of the noise2self paper can be used to select hyper parameters for algorithms using single cell count data. In the context of this work , it can be used to estimate an optimal number of PCs for downstream denoising. For comparison, we used noise2self-MCV to select the optimal number of PCs which are used as input to DEWAKSS, and included it in Figure 4 (labeled DEWAKSS robust).

In addition, we have clarified a few points in the introduction and methods to clearly delineate our application of noise2self from the MCV-based application. We have also repeated the analysis from the authors of noise2self and MCV in figure S.8. with the attached clarifying text in the introduction and in the method section.

**(b) the downstream analysis (variance retained) section is very limited. For instance, I don't follow how the authors use the number of PCs to recapture total variance after denoising as an argument for better performance. Please clarify in text.**

This is a key component of our analysis, so clarity here is critical and we have made substantive changes to help explain the rationale for this analysis. Within gene expression data, some variance is random, and this noise can interfere with or corrupt interpretation. Removing this noise allows for a more informative downstream analysis. Other variance is informative and is the result of underlying biology. Removing too much variation will obfuscate or eliminate biological signals in the data and will result in a highly biased analysis. When this is taken to the extreme, almost all of the variance in gene expression data can be captured in a handful of dimensions. At this extreme, features within the data become very collinear, more complex interactions between features disappear, and only the strongest sources of variance are preserved. All denoising methods must make tradeoffs between removing noise and retaining signal, but when biological data is highly compressed into a handful of dimensions, it is very likely that a considerable amount of biological variance has been removed.

Retaining variance in the denoised data is not an explicit objective of DEWAKSS. Instead DEWAKSS relies on the objective function, constructed using the noise2self principle. This objective is designed to find the “optimal'' hyperparameters given the objective (removing noise). Many methods discard >90% of the variance in a data set. Given our objective function, this is smoothing much more than would be warranted based on the structure of the data. It is not trivial to determine when/if this is useful as it is likely dependent on the heuristics of the smoothing algorithm and the data, and in many cases the transformation is so lossy that we believe it is very unlikely to give a good representation of the data for general downstream analysis.

**(c) a more thorough analysis of differential expression can be performed beyond showing the effect on just a small number of marker genes. An analysis similar to the benchmark from Hou et al. https://www.biorxiv.org/content/10.1101/2020.01.29.925974v1 would be helpful.**

This is a very useful suggestion. We have chosen to use the benchmark based on Hou et al, but have elected to compare expression data from the model organism *Saccharomyces cerevisiae*. We have chosen a yeast single-cell data set with pooled gene deletions that has comparable published bulk experiments from a seminal paper in large-scale perturbation analysis. Differential gene expression comparisons between single-cell and a bulk ‘ground truth’ experiment before and after imputation have been included in section 2.6.

**(d) this is minor, but there is a statement that any kNN graph can be used but no experiment on the effect of the algorithm used to construct the kNN graph is provided. The UMAP method is fairly sophisticated and I wonder how much of the results presented here are based on how well that construction is made. Perhaps, a test with at least one other construction method would be helpful.**

This is a very good point. The UMAP algorithm for finding neighbors is only used if the number of features are above 4096. Otherwise all neighbours are computed and closest k are selected. Several of the datasets in the analysis are not using the UMAP algorithm to find the neighbours. We now made a clarifying statement in the methods section to address this.

**Reviewer #2**

**This is an overall well-structured paper proposing data imputation and denoising in single-cell transcriptomic data. The imputation is based on learning cell-cell similarity. It used the expression of a gene on neighboring cells of the cell of interest to impute the expression level of the same gene on this cell. The authors outlined their computational algorithm and use real scRNA-seq datasets to demonstrate its utility and compared with some existing imputation methods. My comments are as follows:**

**1. In the algorithm description on pages 10 and 11, Equations (2) and (5) have typo. Subscript k does not appear on the right side of both equations.**

This is an important point to clarify, as it is the reason why our denoising function is J-invariant and hence suitable for this form of self-supervised denoising.

In equation (2), we describe denoising via simple matrix multiplication, without enforcing J-invariance. In this situation, the expression of gene $j$ in cell $k$ is calculated using a weighted sum of the expression of gene $j$ in all cells $\hat{k}=1, ... , z$. The summation from $1$ to $z$ includes $\hat{k}=k$.

In equation (5), we wish to show how our method enforces J-invariance and is hence suitable for self-supervised denoising via our objective function, in line with the noise2self framework. We highlight that the expression of gene $j$ in cell $k$ is calculated using a weighted sum of the expression of gene $j$ in all cells except cell $k$. This is why cell $k$ appears on the left hand side of the equation but not on the right hand side. This is what makes the denoising function J-invariant - for a given gene, the value of the denoised expression in a particular cell is independent of the value of the non-denoised expression in that cell.

**2. It is unclear the benefit of setting the diagonal elements of the Markov matrix M to 0. How does this step impact the performance in the real data analysis? Apparently, MAGIC does not require this step. The authors can compare their algorithm performance with and without this step.**

Building the knn graph will by default not set diagonal elements to some nonzero value as each datapoint has a zero distance to itself and is not a neighbour to itself. The starting graph is therefore very similar for DEWAKSS and MAGIC. Setting diagonal elements to zero comes into play once diffusion is done. At that point nodes/cells in the graph can become self referential. Something that breaks the noise2self principle. Therefore we would expect to see a minimum prediction error after 2 iterations when cells can be predictors of themselves. We illustrate this in the added supplemental figure S7 and extend this observation with an explanation in the methods section.

**3. There are many imputation methods available in literature. The authors compared the performance of their method to DrImpute and SAVER in the assessment of cell-cell correlation, and to DeepImpute and MAGIC in cell type clustering. In the last experiment to assess data variance, the authors compared to MAGIC. There is a lack of comprehensive comparisons. I suggest to add a few more existing methods and compare them with the new one in every experiments outlined in this paper.**

To address this issue we choose to make a consistent comparison with the state of the art methods DrImpute and SAVER and add those to all benchmarks. E.g. in figures, 3 and 4 in the current manuscript as well as any of the new analysis in the manuscript.

**4. What is the computation cost? Please benchmark the computation time in comparison to other existing methods.**

That is an excellent question. We performed a computational benchmark of the main 5 methods included in the manuscript and have included it as Figure 6 (with the raw results available in table S1).
