# Supplementary material for: Optimal tuning of weighted kNN- and diffusion-based methods for denoising single cell genomics data
Source: PLoS Comput Biol. 2021 Jan 7;17(1):e1008569. doi: 10.1371/journal.pcbi.1008569 (PMC7817019; doi:10.1371/journal.pcbi.1008569)
Supplement: Attachment — Submitted filename: comments.docx [file pcbi.1008569.s014.docx]

Most of my previous comments were addressed to my satisfaction except my first comment.

In Eq. (1), the matrix M represents cell-to-cell similarity and is a right-stochastic matrix with each row summing to 1. In the proposed new algorithm, the imputation was done by calculating the weighted average of express levels of the same gene in neighboring cells to recover the gene expression in a cell. So in Eq. (2), the imputed value of gene j in cell k is calculated by $\sum_{k^{'}=1}^{z} u_{kk'}x_{k'j}$, where the weight $u_{kk'}$ is the $kk'$-th element of M and represents the similarity between cells $k$ and $k'$. Additionally, the constrain on row sum of M was wrongly presented as column sum in the manuscript. Of course, if M is a square matrix, it would have the same constrains on row sum and column sum. Follow the similar argument, the subscripts in Eq. (5) also contained errors.
