# Supplementary material for: Optimal tuning of weighted kNN- and diffusion-based methods for denoising single cell genomics data
Source: PLoS Comput Biol. 2021 Jan 7;17(1):e1008569. doi: 10.1371/journal.pcbi.1008569 (PMC7817019; doi:10.1371/journal.pcbi.1008569)
Supplement: Attachment — Submitted filename: Response to reviewer (PCOMPBIOL-D-20-00345R1).docx [file pcbi.1008569.s015.docx]

Dear Dr. Qing Nie and Dr. Weixiong Zhang,

Thank you for the careful review of our manuscript. We have addressed in full the final comments and have updated the manuscript accordingly. A copy of the revised manuscript with changes highlighted as well as a clean copy has been attached to this correspondence as requested. The error pointed out by reviewer #2 has been corrected, and we have modified figure 5 to be more intuitive in response to the suggestion by reviewer #1. A detailed response to these points is included below.

Sincerely,

Dr. Richard Bonneau and Dr. Andreas Tjärnberg

--------------

**Reviewer #1:

I appreciate the response from the authors and see all my questions addressed. While I recommend acceptance, better presentation of the new benchmark results would be great.**

Thank you. We agree that figure 5 was not laid out well and have changed it to be more easily interpreted by plotting the metric changes compared to the control. The underlying results are not changed.

**Reviewer #2:**

**Most of my previous comments were addressed to my satisfaction except my first comment.**

**In Eq. (1), the matrix M represents cell-to-cell similarity and is a right-stochastic matrix with each row summing to 1. In the proposed new algorithm, the imputation was done by calculating the weighted average of express levels of the same gene in neighboring cells to recover the gene expression in a cell. So in Eq. (2), the imputed value of gene j in cell k is calculated by , where the weight is the -th element of M and represents the similarity between cells and . Additionally, the constrain on row sum of M was wrongly presented as column sum in the manuscript. Of course, if M is a square matrix, it would have the same constrains on row sum and column sum. Follow the similar argument, the subscripts in Eq. (5) also contained errors.**

We want to thank the reviewer for the persistence in highlighting this error. We have now fully corrected the relevant equations by accurately assigning the cell x cell indices k and khat in the sum and notation.
